# Supplementary figures and images for: Renal antiporter ClC-5 regulates collagen I/IV through the β-catenin pathway and lysosomal degradation
Source: Life Sci Alliance. 2024 Apr 26;7(7):e202302444. doi: 10.26508/lsa.202302444 (PMC11053357; doi:10.26508/lsa.202302444)

FIGURE 1

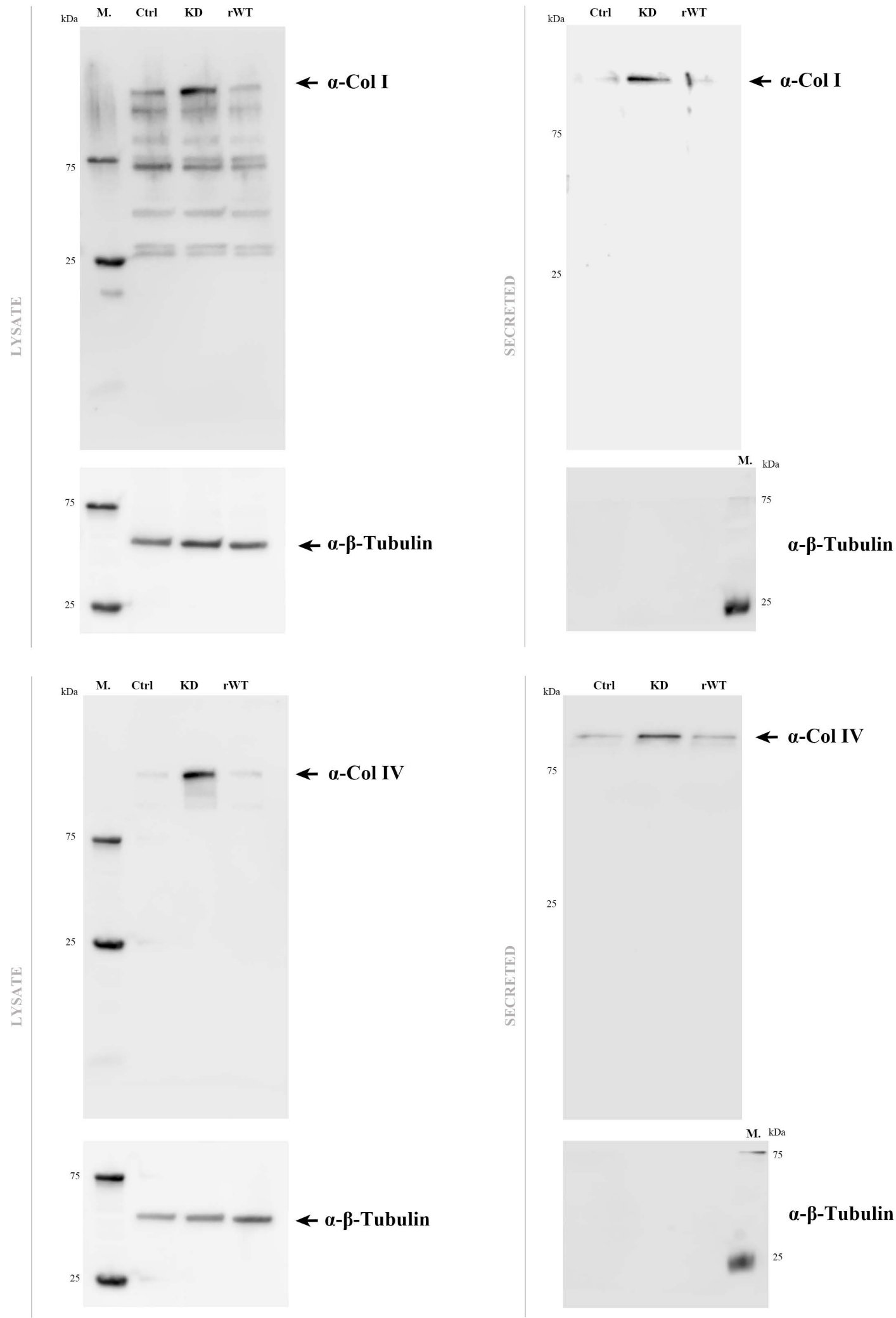

FIGURE 3

A)

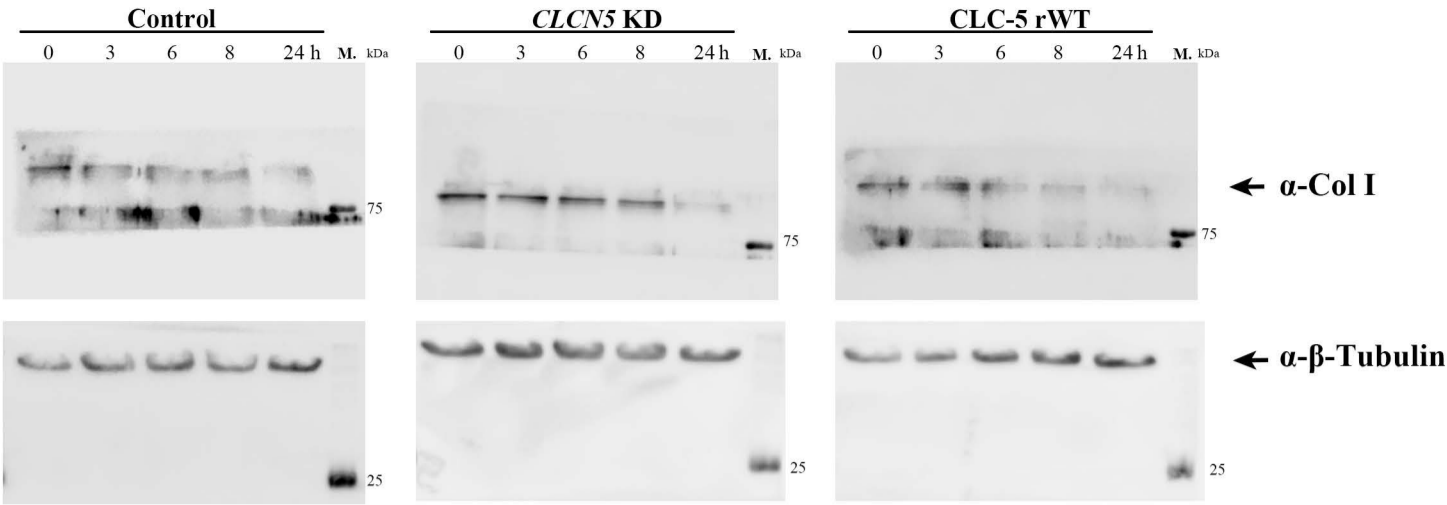

B)

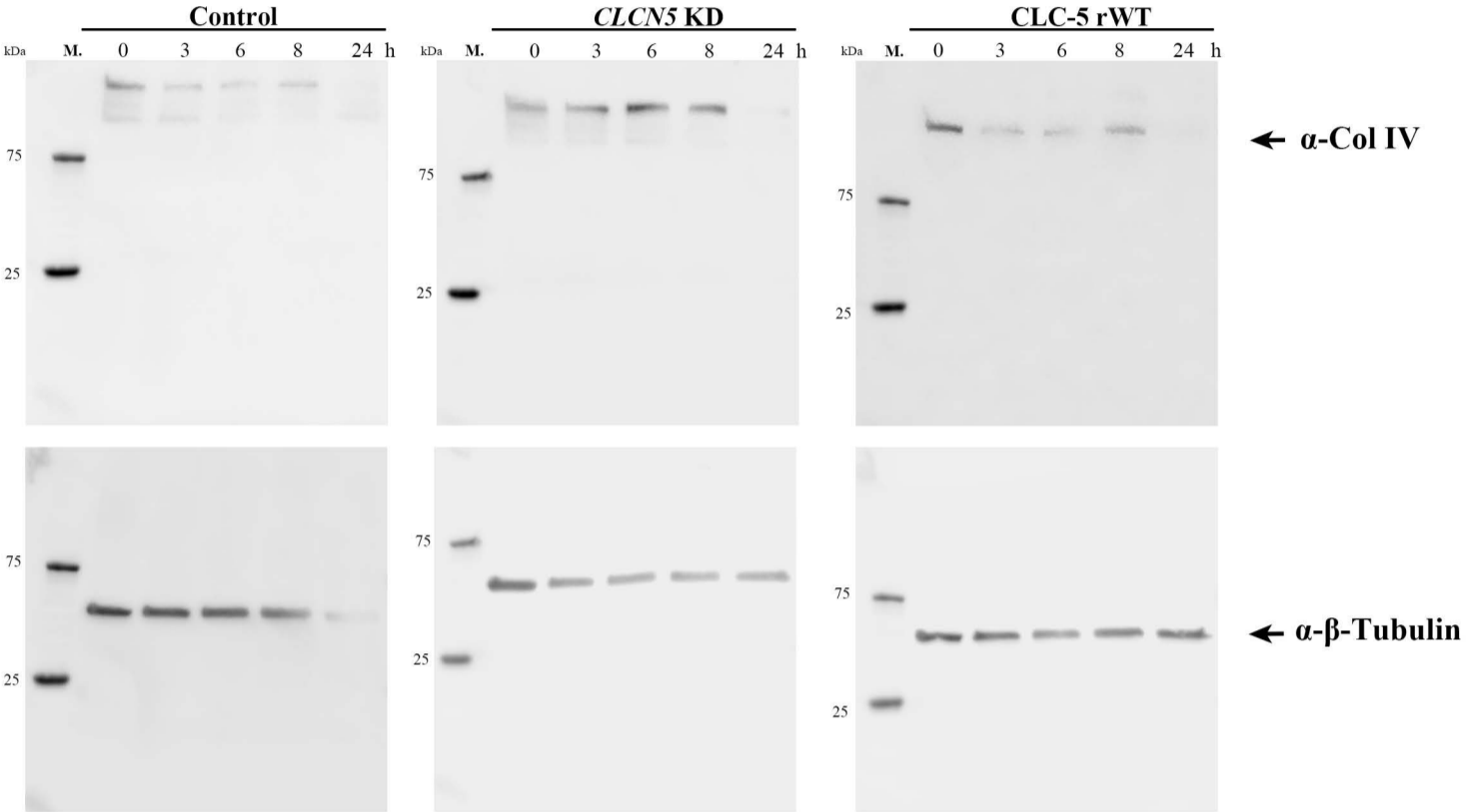

C)

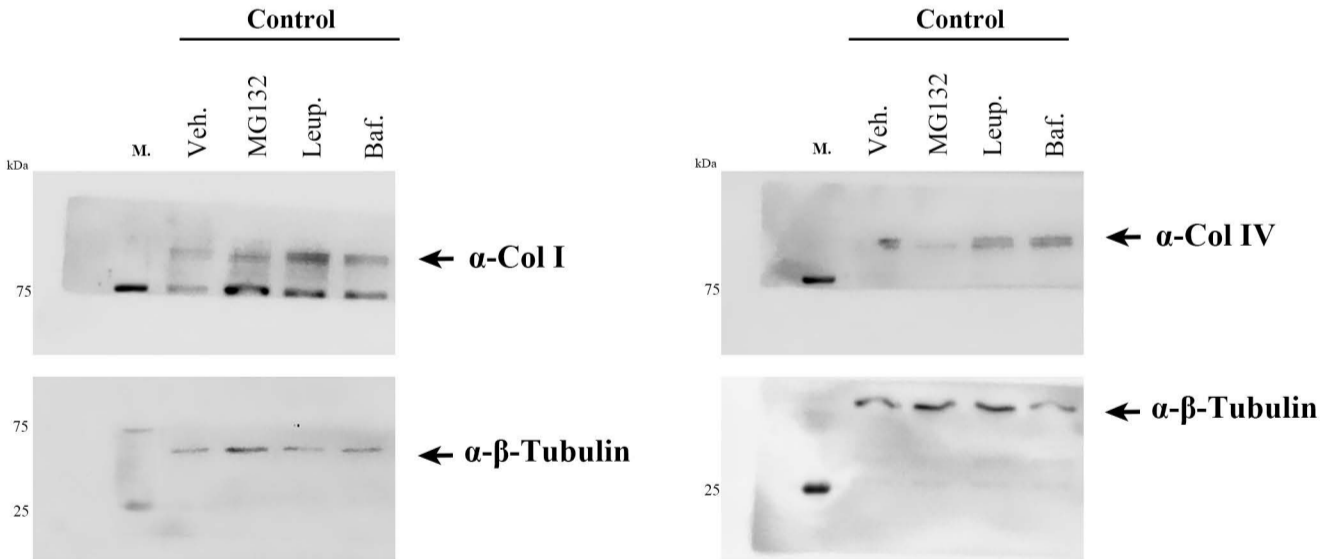

D)

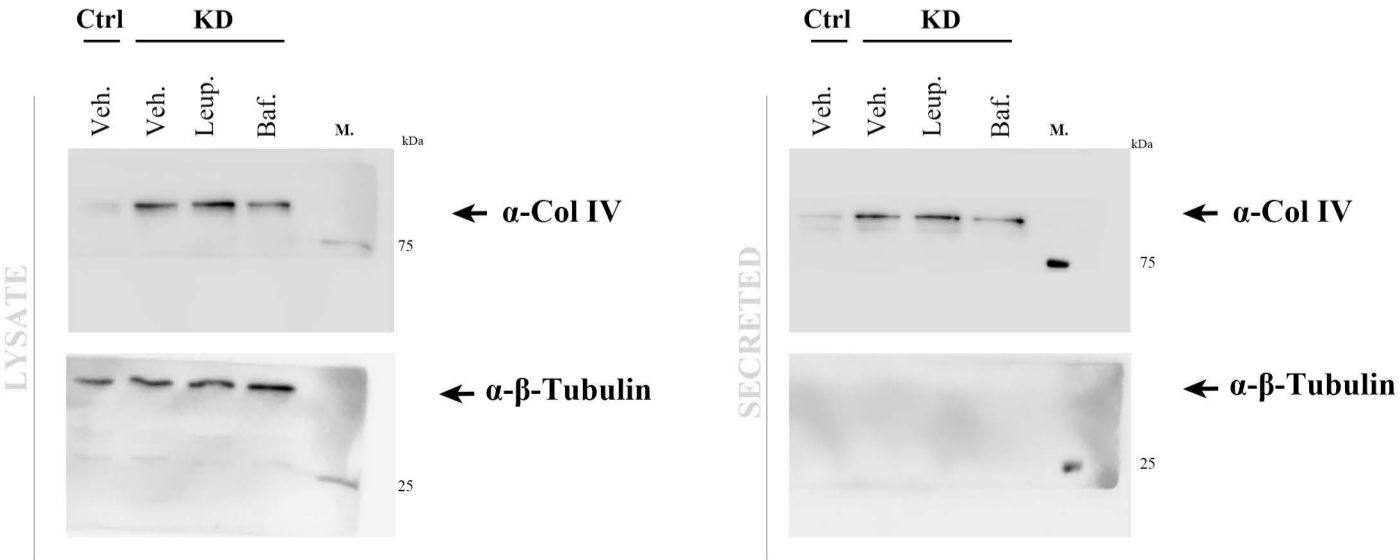

FIGURE 5

D)

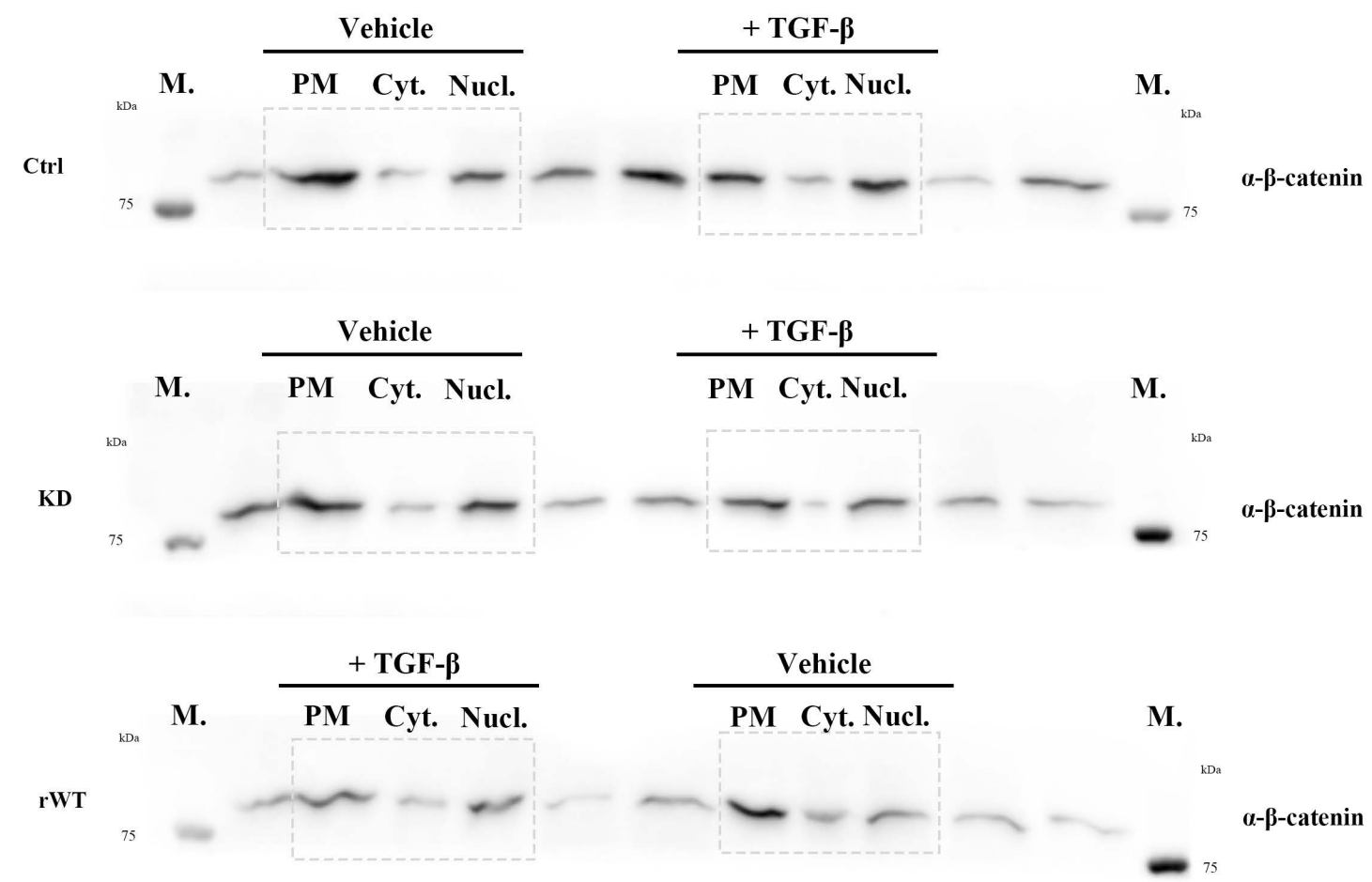

E)

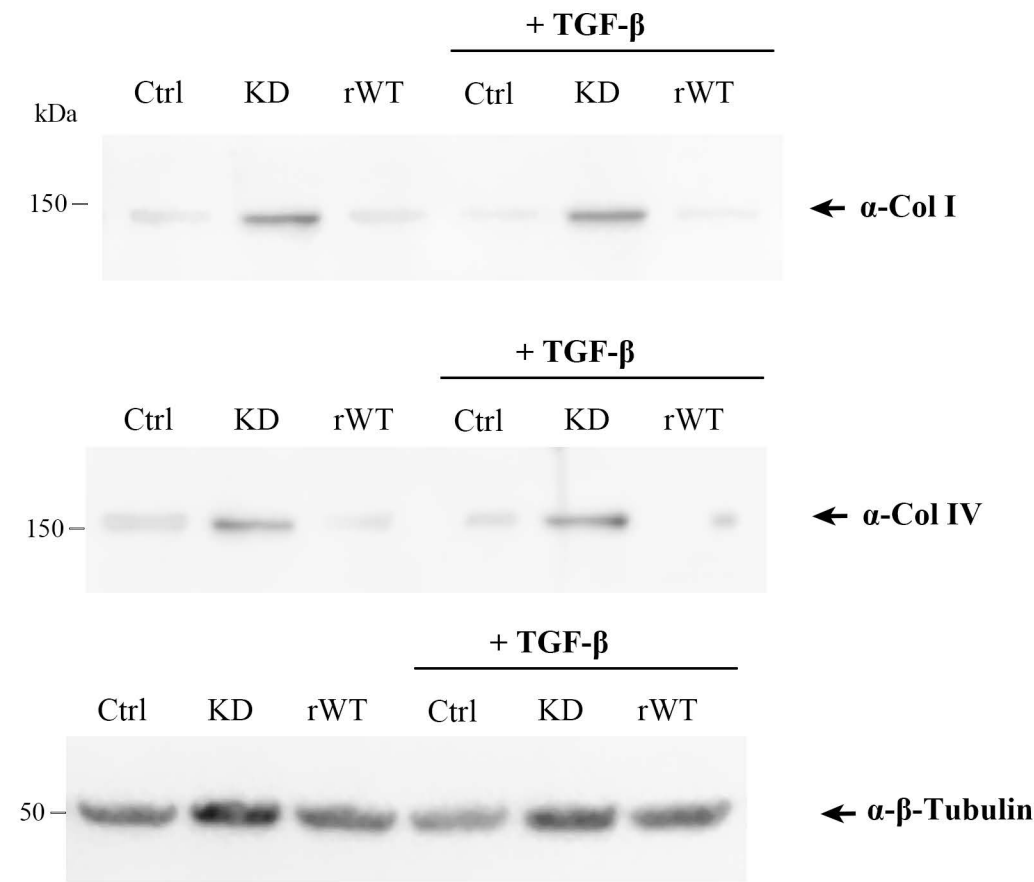

G)

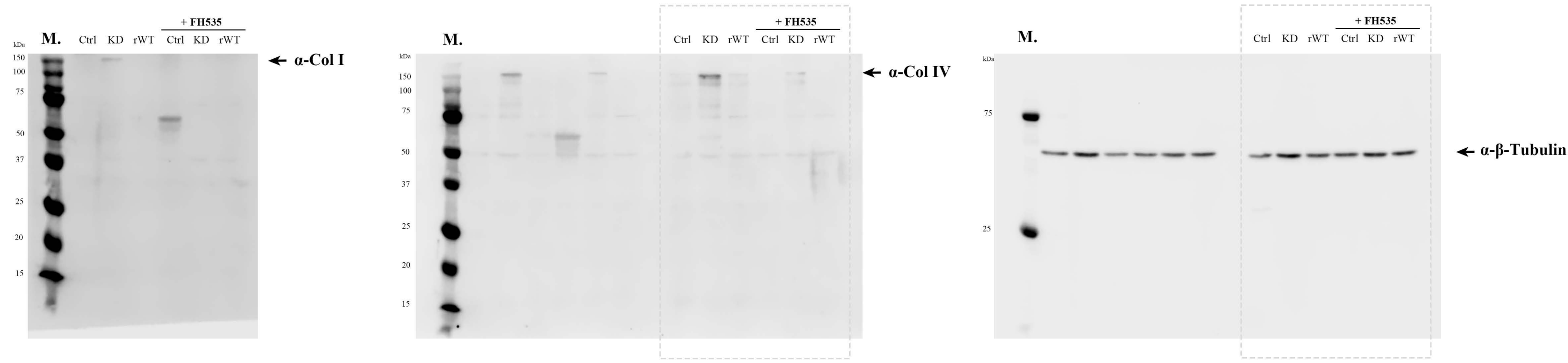

FIGURE 6

A)

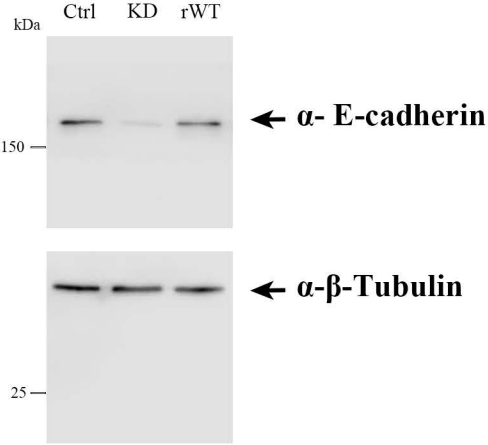

B)

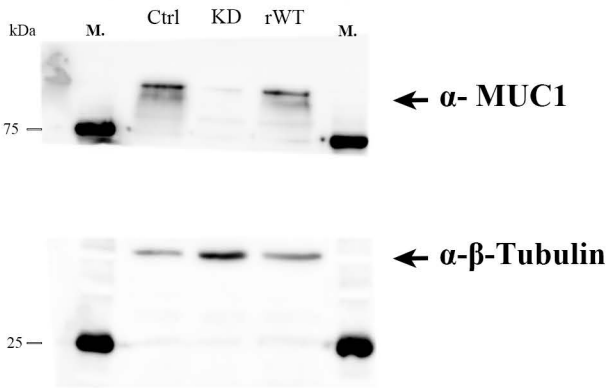

D)

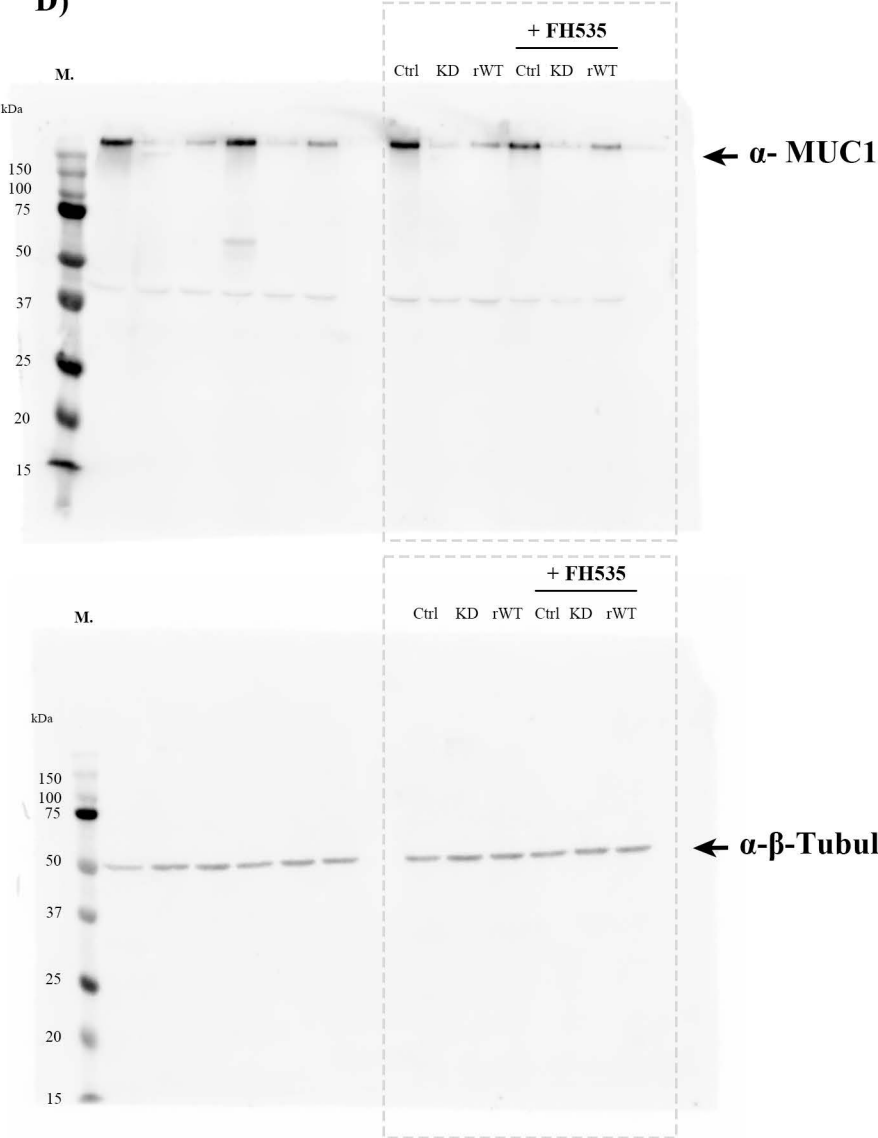

E)

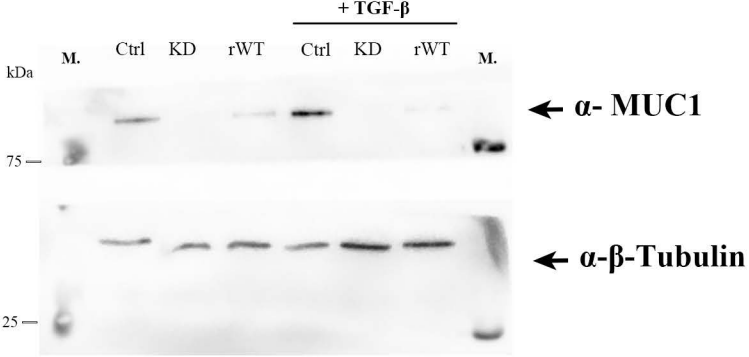

F)

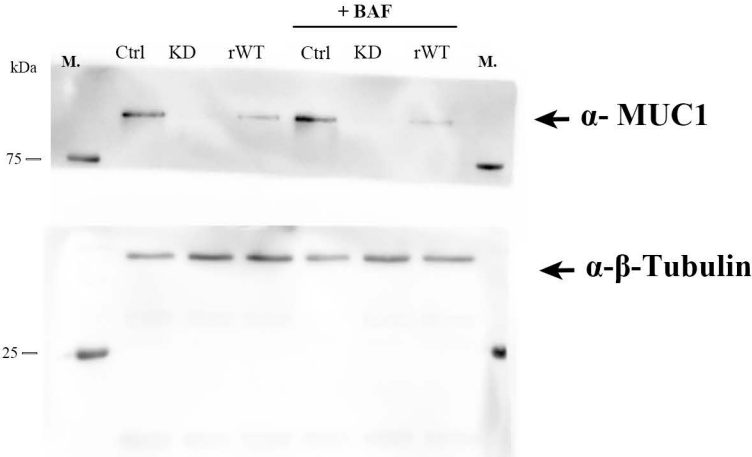

Supplement: Supplementary file 1 [file LSA-2023-02444_SdataF1_F3_F5_F6.pdf]
