# Supplementary material for: Renal antiporter ClC-5 regulates collagen I/IV through the β-catenin pathway and lysosomal degradation
Source: Life Sci Alliance. 2024 Apr 26;7(7):e202302444. doi: 10.26508/lsa.202302444 (PMC11053357; doi:10.26508/lsa.202302444)
Supplement: Supplementary file 2 [file LSA-2023-02444_TableS1.docx]

**Supplementary table 1**

| **Gene** | **Protein** | **Regulation** | **logFC** | **Adj P Val** |  |
| --- | --- | --- | --- | --- | --- |
| **WNT7A** | Protein Wnt-7a | UP | 1.9 | 3.15E-12 |  |
| **SOX2** | Transcription factor SOX-2 | UP | 1.2 | 7.34E-08 |  |
| **EPHB2** | Ephrin type-B receptors | UP | 1.0 | 1.23E-10 |  |
| **WNT7B** | Protein Wnt-7b | UP | 1.0 | 2.77E-07 |  |
| **EDN1** | Endothelin-1 | UP | 0.9 | 3.98E-05 |  |
| **SOX17** | Transcription factor SOX-17 | UP | 0.9 | 8.81E-07 |  |
| **WNT5A** | Protein Wnt-5a | UP | 0.8 | 8.92E-04 |  |
| **ENC1** | Ectodermal-neural cortex 1 | UP | 0.8 | 6.92E-06 |  |
| **FGF18** | Fibroblast growth factor 18 | UP | 0.7 | 7.59E-04 |  |
| **TNFRSF11B** | Tumor necrosis factor receptor superfamily member 11B / Osteoprotegerin | UP | 0.7 | 9.57E-04 |  |
| **LBH** | Protein LBH | UP | 0.6 | 2.94E-03 |  |
| **ITF2 (TCF4)** | Immunoglobulin transcription factor 2 | UP | 0.6 | 3.67E-03 |  |
| **BMP4** | Bone morphogenetic protein 4 | UP | 0.6 | 1.05E-03 |  |
| **CCND1** | Cyclin-D1 | UP | 0.6 | 1.40E-03 |  |
| **PTTG** | Securin | UP | 0.5 | 4.53E-02 |  |
| **MYC** | Myc proto-oncogene protein | UP | 0.5 | 7.23E-03 |  |
| **CDKN2A** | p16INK4a | UP | 0.4 | 4.98E-03 |  |
| **FZD7** | Frizzled 7 | UP | 0.4 | 7.56E-03 |  |
| **FGF9** | Fibroblast growth factor 9 | UP | 0.3 | 3.18E-02 |  |
| **RUNX2** | Runt-related transcription factor 2 | UP | 0.3 | 3.46E-02 |  |
| **SOX9** | Transcription factor Sox-9 | DOWN | -0.5 | 1.75E-02 |  |
| **EFNB1** | Ephrin-B1 | DOWN | -1.4 | 1.26E-09 |  |
