# Supplementary material for: Renal antiporter ClC-5 regulates collagen I/IV through the β-catenin pathway and lysosomal degradation
Source: Life Sci Alliance. 2024 Apr 26;7(7):e202302444. doi: 10.26508/lsa.202302444 (PMC11053357; doi:10.26508/lsa.202302444)
Supplement: Supplementary file 3 [file LSA-2023-02444_TableS2.docx]

**Supplementary table 2**

| Genotype | **Haematoxylin & eosin (H/E) staining** | | | | |
| --- | --- | --- | --- | --- | --- |
|  | Epithelial hyperplasia | Inflammatory infiltrate | Oedema | Structure glomeruli | Structure tubules |
| **Clcn5^+/+^** | N | N | 0 | N | N |
| **Clcn5^+/-^** | N | N | 0 - 1 | N | N |
| **Clcn5^-/-^** | N | N | 0 - 1 | N | N |

N: Normal; Score Oedema: 0: normal, 1: minimal
